# Nutrition in Cystic Fibrosis: as important as the management of pulmonary disease

**DOI:** 10.1016/j.rpped.2014.12.001

**Published:** 2015-03

**Authors:** Fabíola Villac Adde

**Affiliations:** Universidade de São Paulo, São Paulo, SP, Brazil

In recent decades, the survival of patients with cystic fibrosis (CF) has increased significantly, with a current median of approximately 38 years in European and North American countries.^1^^,^ 2 Currently, almost 50% of CF patients in the United States are adults.^2^ A better approach in the treatment of pulmonary disease and improvement of patients' nutritional status were responsible for these changes. 

In the 80s, Corey et al, comparing two similar groups of patients with CF followed in Boston and Toronto, observed increased survival in the group from Canada (30 *versus* 21 years).^3^ The better nutritional status of these patients was the determining factor for this difference, as the degree of pulmonary involvement was comparable in both groups. While patients from Toronto received a diet without restriction of fat, the ones from Boston received a low-fat diet and, therefore, one with lower caloric intake. It was concluded that although the progressive pulmonary disease is the leading cause of mortality in CF, concerns with nutritional guidance and intervention in these patients was essential. 

Many studies have suggested that severe pulmonary disease is consistently correlated with nutritional status deterioration and, on the other hand, prevention of malnutrition is related to a better course of pulmonary disease and longer patient survival.^4^^,^ 5 Several authors have shown that patients undergoing aggressive nutritional rehabilitation through high-calorie diets by gastrostomy or nasogastric tube, improved their nutritional status, while pulmonary disease was stabilized.^6^^,^ 7

A national study, evaluating 87 patients with CF aged 6 months to 18 years and grouping them according to overall disease severity measured by the Shwachman score (CF severity score) also showed that patients with less pulmonary involvement had better nutritional status and those with increased pulmonary involvement showed poor nutritional status, with significant differences between the groups regarding the different anthropometric variables assessed. The use of simple measures of nutritional advice in the long term allowed attaining greater adherence to enzyme supplementation and high-calorie dietary supplements, contributing to nutritional status improvement observed in some of these patients.^8^

Hortencio et al, in an article published in this issue of the journal and that evaluated 52 patients with a mean age of 6 years, whose CF diagnosis was made at a median close to two years, found 40.4% of patients with Z BMI / A scores <-2 and 38% with H/A score <-2 at the first visit to the referral center, which reflected severe nutritional impairment. With the implementation of therapeutic measures - enzyme supplementation, hypercaloric diet and pulmonary disease management - most patients showed nutritional recovery, with a decrease in BMI/A and H/A Z scores <-2 to 7.7% (only 4 patients) in both indices. However, as the authors emphasize, patients with BMI/A and H/A Z scores between -1 and -2 must be considered at nutritional risk and this was verified in 23 and 30.8% of patients in this study, respectively.

This sentence group of patients must undergo stringent monitoring of nutritional status. Some associations between better anthropometric indices and lower severity of respiratory disease were observed as fewer hospitalizations, later respiratory symptom onset and better results on spirometry. However, the finding of a better H/A index with longer time between birth and diagnosis and between the first visit and diagnosis was paradoxical, as 90.8% of patients were pancreatic insufficient and in this situation, one would expect a worsening in this anthropometric index with diagnostic delay and thus, at the start of pancreatic enzyme supplementation.^9^

With the implementation of newborn screening for CF in the state of São Paulo in February 2010 10 we expect to attain an early diagnosis of CF, allowing timely therapeutic interventions to be made and attaining benefits such as improved growth, nutrition, pulmonary function and prognosis, as observed in countries where newborn screening for CF is already a routine.^11^^,^ 12
