# Supplementary material for: Nutrition in Cystic Fibrosis: as important as the management of pulmonary disease
Source: Rev Paul Pediatr. 2015 Mar;33(1):1–2. doi: 10.1016/j.rpped.2014.12.001 (PMC4436949; doi:10.1016/j.rpped.2014.12.001)
Supplement: Translation

# Translation: Nutrição em fibrose cística: tão importante quanto o manejo da doença pulmonar

**Authors:** Fabíola Villac Adde

**Affiliations:** Universidade de São Paulo (USP), São Paulo, SP, Brasil

Nas últimas décadas, a sobrevida dos pacientes com fibrose cística (FC) aumentou expressivamente, com mediana atual de 38 anos em países europeus e americanos.^1^ ^and^ 2 Atualmente, quase 50% dos pacientes com FC nos Estados Unidos são adultos.^2^ A melhor abordagem no tratamento da doença pulmonar e a melhoria do estado nutricional desses pacientes foram os grandes responsáveis por essas mudanças.

Já na década de 1980, Corey et al., comparando dois grupos semelhantes de pacientes com FC acompanhados em Boston e Toronto, observaram maior sobrevida no grupo do Canadá (30 *versus* 21 anos). 3 O melhor estado nutricional desses pacientes foi o fator determinante dessa diferença, pois o grau de acometimento pulmonar era comparável nos dois grupos. Enquanto os pacientes de Toronto recebiam dieta sem restrição de gordura, os de Boston recebiam dieta hipogordurosa e, portanto, com menor aporte calórico. Concluiu-se, que apesar de a doença pulmonar progressiva ser a maior causa de mortalidade na FC, a preocupação na orientação e na intervenção nutricional desses pacientes era fundamental.

Muitos estudos sugerem que a doença pulmonar grave se correlaciona consistentemente com a pioria do estado nutricional e que, por outro lado, a prevenção da desnutrição está relacionada com melhor curso da doença pulmonar e maior sobrevida do paciente.^4^ ^and^ 5

Diversos autores mostraram que pacientes submetidos a reabilitação nutricional agressiva, por meio de dietas hipercalóricas por gastrostomia ou sonda nasogástrica, melhoraram o estado nutricional e ao mesmo tempo estabilizaram a doença pulmonar.^6^ ^and^ 7

Estudo nacional, que avaliou 87 pacientes com FC de seis meses a 18 anos e os agrupou de acordo com a gravidade geral da doença, medida pelo escore de Shwachman (escore de gravidade da FC), também mostrou que pacientes com menor acometimento pulmonar apresentavam melhor estado nutricional e aqueles com maior acometimento pulmonar encontravam-se muito comprometidos do ponto de vista nutricional. As diferenças entre os grupos foram significativas nas diversas variáveis antropométricas avaliadas. Com medidas simples de aconselhamento nutricional em longo prazo, foi possível obter-se maior adesão à suplementação enzimática e ao uso de suplementos dietéticos hipercalóricos, o que contribuiu para a melhoria do estado nutricional observada em uma parcela desses pacientes.^8^

Hortencio et al., em artigo publicado nesta edição da revista e que avaliou 52 pacientes com média de seis anos cujo diagnóstico de FC ocorreu em mediana próximo aos dois anos, encontraram 40,4% dos pacientes com escore Z IMC/I <-2 e 38% com escore Z A/I <-2 na primeira consulta no centro de referência, o que refletiu grave acometimento nutricional. Com a implementação das medidas terapêuticas - suplementação enzimática, dieta hipercalórica e manejo da doença pulmonar - houve recuperação nutricional na maioria dos pacientes e caíram os valores dos escores Z IMC/I e Z A/I abaixo de -2 para 7,7% (apenas quatro pacientes) em ambos os índices. Porém, como os próprios autores frisam, pacientes com escores Z de IMC/I e de A/I entre -1 e -2 devem ser considerados em risco nutricional e isso ocorreu em 23% e 30,8% dos pacientes desse estudo, respectivamente. Esse grupo de pacientes merece vigilância extrema do estado nutricional. Algumas relações entre melhores índices antropométricos e menor gravidade da doença respiratória foram constatadas, como menos internações, manifestações respiratórias mais tardias e melhores índices na espirometria. Entretanto, foi paradoxal o encontro de relação de melhor índice de A/I com maior tempo tanto entre o nascimento e o diagnóstico quanto entre a primeira consulta e o diagnóstico, uma vez que 90,8% dos pacientes eram pancreato-insuficientes e, nessa situação, esperar-se-ia uma pioria nesse índice antropométrico com o retardo no diagnóstico e, portanto, no início da suplementação das enzimas pancreáticas.^9^

Com a implantação da triagem neonatal para FC no Estado de São Paulo em fevereiro de 2010^10^ espera-se que tenhamos o diagnóstico precoce da FC, o que permitirá intervenções terapêuticas em tempo oportuno e se obterem benefícios como melhor crescimento, nutrição, função pulmonar e prognóstico, como observado nos países onde a triagem neonatal para FC já é rotineira.^11^ ^and^ 12
